# Notes on the Use of Bacteriological Methods of Diagnosis in Private Practice.—I

**Published:** 1898-09-17

**Authors:** J. O. Symes

**Affiliations:** Bacteriologist Bristol Royal Infirmary


### Medical Progress and Hospital Clinics.

[*The Editor will be glad to receive offers of co-operation and contributions from members of the profession. All letters should be addressed to* The Editor, at the Office, 28 & 29, Southampton Street, Strand, London, W.C.]

### NOTES ON THE USE OF BACTERIOLOGICAL METHODS OF DIAGNOSIS IN PRIVATE PRACTICE.—I.

By J. O. Symes, M.D., D.P.H.Lond., Bacteriologist Bristol Royal Infirmary.

At the present time, when bacteriological methods are being increasingly employed in the solution of the various problems of diagnosis, there has perhaps been in some quarters a tendency to lay too great a stress upon bacteriological data, and to disparage patient and accurate clinical observation. This is to be regretted, for the science of bacteriology has no more claim to infallibility than has any other department of the medical art. The finding of bacteriology is only to be regarded as a link in the chain of evidence which is built up from a series of clinical observations.

Unfortunately, the pursuit of bacteriological research necessitates the provision of elaborate apparatus, incubator, steriliser, culture-media, &c. Moreover, a special room must be provided, and the time required for the work is more than a man in general practice can be expected to give. Consequently, the practitioner has been deprived to a great extent of much of the valuable help which is available to his more favoured brethren who are attached to one of the numerous large teaching institutions. More recently, however, institutes, associations, public and private laboratories, all engaged in the work of bacteriological research, have been founded in all parts of the kingdom, and to these specimens may be sent for examination and a speedy report obtained. Morbid products sent by post must be placed in a hermetically sealed receptacle, such as a bottle or test tube, tightly corked and sealed. Absorbent material must be wrapped round the vessel, and the whole packed in a wooden, leather, or metal case. The postal regulations also require that the package shall be handed in at the post office, registered, and not transmitted through the parcel post.

Although the opportunities of obtaining bacteriological examinations have thus been facilitated, it cannot be said that the advantage has been made use of as fully as could be desired. This is in part due to a lack of knowledge as to how best to take specimens for examination, and what cases are suitable for such investigation, and in part to the want of the necessary apparatus at the moment the cases present themselves.

It is the purpose of the present articles to point out the diseases in which help may be obtained by bacteriological methods, to enumerate the few simple necessaries required in taking specimens, and to indicate the way in which these instruments should be employed.

With regard to instruments, the first essential is a microscope with a lens of a sixth, or preferably an eighth, of an inch focus. With such a lens really useful work may be done, but it would, of course, be preferable to use a one-twelfth oil immersion, which can now be obtained from Leitz or Beck for a sum of about £5. No. 1 cover-slips should be first boiled in nitric acid, washed in distilled water, and kept in absolute alcohol.

For the reception of pus, sputum, urine, diphtheritic membrane, or serous effusions a test tube or small bottle may be utilised; the small glass cylinders in which samples of compressed drugs are sent are also useful for this purpose. The vessel should be first sterilised by lengthened boiling with soda, and the cork completely enveloped in oil-silk (boiled), so that the contents may not be contaminated.

For obtaining small quantities of material to smear on cover-slips, or for inoculating culture tubes, a wire is most convenient. It is best made of platinum, should be three or four inches long, with a small loop at the free end, and may be mounted on either a glass or wooden holder. An equally efficient instrument may be improvised from a piece of copper wire six inches long, one end of which may be beaten out flat, whilst the other is roughened to permit a swab of cotton wool being wrapped around it. A silver probe may be employed, or any other such instrument, provided only that it is capable of being sterilised by heating in the flame of a spirit-lamp.

For collecting specimens of blood, as required for the serum diagnosis of enteric fever, &c., the ordinary capillary vaccine tubes are available. They should first be slowly passed two or three times through the flame of a spirit-lamp.

In some few cases it is a great advantage to transfer the morbid products directly to the culture medium, and for this reason it is well to be provided with tubes for inoculation. Blood serum tubes are the most generally useful, and should be obtained two or three at a time from any of the bacteriological laboratories or wholesale chemists. If kept for any lengthened period of time they are apt to become dry and useless.

For microscopic work the most useful all-round stain is Löffler's methylene blue (saturated solution of methylene blue in alcohol, 30 c.c., solution of potassium hydrate in distilled water, 1-10,000, 100 c.c.). Special stains are required for the detection of tubercle bacilli, and these will be spoken of under the head of tuberculosis.

Having now enumerated the necessary materials, the next step is to describe the method of their use. The diseases which will be touched upon in these papers are those in which bacteriological methods of diagnosis have proved of most utility, viz., acute and chronic suppuration, diseases of serous membranes, general infections, tuberculosis, diphtheria and allied throat affections, and enteric fever. Under the head of the several diseases will be described in detail the steps to be taken in order to procure cultures or coverglass preparations of the micro-organisms associated with them.

**Suppuration.**—Practically all cases of acute suppuration are produced by the action of living micro-organisms, the commonest varieties being the staphylococci, streptococci, bacillus coli communis, and pneumococcus. The finding of one or other of these organisms in a pus may have an important bearing on diagnosis, prognosis, and treatment. For instance, in a wound, whilst the marked presence of streptococci would suggest a diffuse phlegmonous condition, with the possibility of secondary infection of the lymphatics, a septicæmia; the finding of staphylococci would be more suggestive of localised suppuration and the pyæmic state. In intra-abdominal suppuration bacillus coli would point to a possible bowel lesion; cocci to a wound of the abdominal parietes; and pneumococci to the lung disorder. An abscess, the pus from which after staining showed no micro-organisms, might, after scraping, be sewn up, the probability being that it is tuberculous; if, on the other hand, cocci are found, this would point to the necessity for drainage. In the case, then, of an unopened abscess the steps to secure a bacteriological examination are as follows:—

The skin is rendered aseptic by scrubbing with soap and water, rubbing with ether or turpentine to rid of fat, soaking in carbolic acid lotion, and finally cleansing with absolute alcohol. The incision being made, the first flow of pus is rejected, and then a drop taken on the wire or needle and spread over a cover-glass. This is fixed by passing through the flame of a spirit lamp two or three times, and may then be stained with methylene blue. The film will require about ten minutes to stain, but if heated over a flame the process is much accelerated. It is then washed in water, and is ready for examination under the microscope.

If culture tubes are available the sterilised needle is dipped into the pus and then lightly passed over the surface of the medium, care being taken not to spread the material too thickly. For obtaining a quantity of pus the mouth of the test tube or bottle (sterilised) is dipped into the stream and as much as possible collected. The bottle is then corked, and is ready for transmission. If the quantity be too small to collect in this way, a little may be sucked up into a piece of sterilised glass tubing, which is then sealed by heating the ends in a flame. It is never sufficient simply to take cultures from pus, cover-glass preparations should always be taken at the same time. In this way accidental contamination of the culture tubes may be detected, and micro-organisms found which, owing to the fact that they are unable to grow on the medium provided, might otherwise have escaped observation. The search for pyogenic organisms has become a matter of considerable importance since the introduction of the treatment of septicæmic conditions by antistreptococcic serum.

The disappointing results which have followed this treatment in many cases have undoubtedly been owing to the fact that the disease has not been due to the streptococcus, but to some other organism. Such mistakes would, in many cases, be obviated by a systematic bacteriological examination of the blood, pus, or serous effusions.

Most valuable for the purpose of diagnosis is the bacteriological examination of pus in the case of discharges from the genito-urinary tract. Early in an attack of gonorrhœa, that is in the first two or three days, the detection of the gonococcus is a comparatively easy matter, and the disease is thus differentiated from a simple urethritis. At a later stage so many extraneous organisms are present that it is a difficult matter to demonstrate the diplococcus, and this difficulty becomes accentuated when we have to examine the material from an old-standing chronic case. To establish the diagnosis of gonorrhœa, it should be possible to demonstrate microscopically the existence of bean-shaped cocci, in groups of 8, 16, or 32, within the pus-cells, such cocci being completely decolorised by Gram's stain.

To obtain specimens, the genital organs are first washed, and pus squeezed from the urethra, the first drop rejected, while the second is received upon a wire and spread over a series of cover-slips. The cover-glasses are then passed through the flame and stained for a quarter of an hour in methylene blue. The micro-organisms, if present, are easily distinguishable within the pus-cells, and the diagnosis is confirmed if they are decolorised by Gram.

The isolation of the gonococcus on culture media is so difficult a task that the procedure will not be discussed here. Gonococci may require to be looked for in many conditions—conjunctivitis, arthritis, abscesses in connection with the genital tract, cystitis, salpingitis, and peritonitis. In women specimens are best taken from the urethra and cervix uteri, as it is in these places that the gonococci are most abundant.

In gleet, where the discharge may be limited to a drop of sero-purulent fluid in the morning, the patient may be directed to take the specimen on a cover-slip, upon which a second is immediately placed, so as to insure a thinly-spread layer of material. The cover-slips are then allowed to dry, and the sample is ready for forwarding.

(*To be continued.*)

